# EEG Biofeedback Decreases Theta and Beta Power While Increasing Alpha Power in Insomniacs: An Open-Label Study

**DOI:** 10.3390/brainsci13111542

**Published:** 2023-11-02

**Authors:** Huicong Wang, Yue Hou, Shuqin Zhan, Ning Li, Jianghong Liu, Penghui Song, Yuping Wang, Hongxing Wang

**Affiliations:** 1 Department of Neurology, Xuanwu Hospital, Capital Medical University, Beijing 100053, China; 15652523402@163.com (H.W.); houyuechina@sina.cn (Y.H.); shqzhan@hotmail.com (S.Z.); lining945110@163.com (N.L.); liujh@xwhosp.org (J.L.); songpenghui0104@163.com (P.S.); 2 Beijing Key Laboratory of Neuromodulation, Beijing 100053, China; 3 Center of Epilepsy, Beijing Institute for Brain Disorders, Capital Medical University, Beijing 100053, China; 4 Center for Sleep and Consciousness Disorders, Beijing Institute for Brain Disorders, Beijing 100053, China; 5 Collaborative Innovation Center for Brain Disorders, Capital Medical University, Beijing 100053, China; 6 Hebei Hospital of Xuanwu Hospital, Capital Medical University, Shijiazhuang 050030, China; 7 Neuromedical Technology Innovation Center of Hebei Province, Shijiazhuang 050030, China

**Keywords:** biofeedback, insomnia, EMG, EEG, alpha power

## Abstract

Insomnia, often associated with anxiety and depression, is a prevalent sleep disorder. Biofeedback (BFB) treatment can help patients gain voluntary control over physiological events such as by utilizing electroencephalography (EEG) and electromyography (EMG) power. Previous studies have rarely predicted biofeedback efficacy by measuring the changes in relative EEG power; therefore, we investigated the clinical efficacy of biofeedback for insomnia and its potential neural mechanisms. We administered biofeedback to 82 patients with insomnia, of whom 68 completed 10 sessions and 14 completed 20 sessions. The average age of the participants was 49.38 ± 12.78 years, with 26 men and 56 women. Each biofeedback session consisted of 5 min of EMG and 30 min of EEG feedback, with 2 min of data recorded before and after the session. Sessions were conducted every other day, and four scale measures were taken before the first, fifth, and tenth sessions and after the twentieth session. After 20 sessions of biofeedback treatment, scores on the Pittsburgh Sleep Quality Index (PSQI) were significantly reduced compared with those before treatment (−5.5 ± 1.43,t = −3.85, *p* = 0.006), and scores on the Beck Depression Inventory (BDI-II) (−7.15 ± 2.43, t = −2.94, *p* = 0.012) and the State-Trait Anxiety Inventory (STAI) (STAI-S: −12.36 ± 3.40, t = −3.63, *p* = 0.003; and STAI-T: −9.86 ± 2.38, t = −4.41, *p* = 0.001) were significantly lower after treatment than before treatment. Beta and theta power were significantly reduced after treatment, compared with before treatment (F = 6.25, *p* = 0.014; and F = 11.91, *p* = 0.001). Alpha power was increased after treatment, compared with before treatment, but the difference was not prominently significant (*p* > 0.05). EMG activity was significantly decreased after treatment, compared with before treatment (F = 2.11, *p* = 0.015). Our findings suggest that BFB treatment based on alpha power and prefrontal EMG relieves insomnia as well as anxiety and depression and may be associated with increased alpha power, decreased beta and theta power, and decreased EMG power.

## 1. Introduction

Insomnia has significant long-term health consequences [1], with prevalence ranging from 4–36% among teens to 9–50% among adults [2], and comorbid insomnia-related conditions such as depression and anxiety are common in patients [3]. The American Academy of Sleep Medicine clinical practice guidelines and the European guidelines for treating insomnia recommend cognitive behavioral therapy (CBT-I) as the first-line treatment for chronic insomnia in adults [4]. If CBT-I is unavailable or ineffective for adult insomnia, benzodiazepines (BZDs), non-BZD receptor agonists, melatonin receptor agonists, antidepressants, and antipsychotics should be considered. However, these drugs will produce side effects [5], emphasizing the need for more non-pharmaceutical interventions for insomnia.

The disruption of hyperarousal, cortical activation, cognition, and somatic dysfunction are common clinical features of insomnia [6]. Hyperarousal can be detected by elevated cortisol levels, heightened muscle tension, high heart rate (HR) variability (HRV), and self-reporting. Increased high-frequency electroencephalography (EEG) activity (beta and gamma), decreased delta activity, and increased rapid eye movement EEG in states of excessive arousal are EEG indicators [7]. Therefore, changing the above indicators through different interventions is not only expected to have therapeutic effects on insomnia but can also be used to monitor the effectiveness of the interventions.

Biofeedback (BFB) is a non-invasive behavioral therapy that helps patients gain voluntary control over physiological events. Previous research has shown that BFB treatment can benefit disorders such as epilepsy [8], migraine [9], stroke [10], chronic insomnia [11,12], anxiety [13], attention-deficit/hyperactivity disorder [14,15], autism spectrum disorder [16], and major depression [17]. As a BFB method, neurofeedback training allows individuals to independently adjust specific brain activities, thereby changing their cognitive functions [18]. BFB is based on the idea that autonomic responses may be conditioned by instrumentation and includes biological monitors and sensors such as electromyography (EMG), EEG, electrodermal activity, skin temperature, HR, HRV, and end-tidal carbon dioxide [19]. Relative power may be a more stable and sensitive method for detecting non-rapid eye movement EEG signals in patients with insomnia [20]. Alpha signals are observed when a person is awake, calm, prepared, meditating, or relaxed [21]. Increasing alpha power can reduce symptoms of anxiety and depression [22] and improve working and episodic memory [23]. Increased forehead muscle tone is considered a sign of psychoemotional tension or stress [24]. EMG feedback reduces muscle tension and arousal associated with some types of insomnia and promotes sleep onset [11]. Initial pressure in insomniacs correlated positively with improved sleep, based on EMG findings [25]. In fact, some patients with insomnia can be treated using BFB to reduce muscle tension in the center of the forehead [26]. Therefore, the combination of the increased alpha frequency band of EEG and decreased frontal EMG power caused by BFB treatment can further increase the effectiveness of BFB in treating insomnia.

Previous studies have not analyzed the changes in EEG relative power after BFB treatment to assess its effectiveness. Therefore, we hypothesized that by increasing EEG alpha BFB treatment in insomnia patients, other frequency bands of brain power could be altered to achieve relief. We evaluated the clinical effectiveness of BFB treatment using the Pittsburgh Sleep Quality Index (PSQI) to measure insomnia and the Beck Depression Inventory (BDI-II) and State-Trait Anxiety Inventory (STAI) to measure emotional status. We analyzed the related results of EEG and frontal EMG power by monitoring the relative power before and after the BFB treatment. This study also explored possible BFB treatments to improve clinical understanding of this therapy and provides a reference for clinical practice.

## 2. Methods

### 2.1. Participants

**Figure 1:**
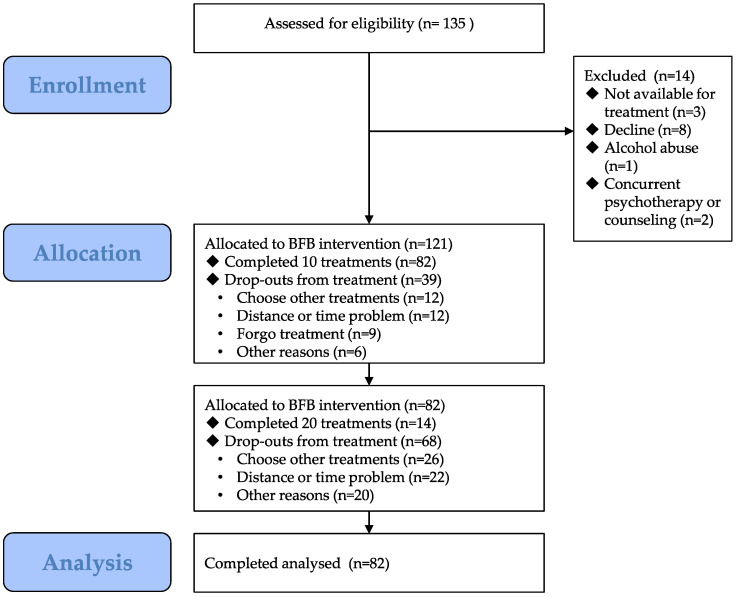
Flow diagram of the study. BFB, biofeedback.

We recruited 135 right-handed patients with primary insomnia from the Department of Neurology, Xuanwu Hospital, Capital Medical University, between 2014 and 2023, and excluded 14 patients who did not meet the inclusion criteria. A total of 121 patients with insomnia were treated using BFB. During this treatment, 82 patients completed more than 10 BFB sessions and 14 completed more than 20 sessions (Figure 1). Patients with incomplete data and those receiving fewer than 10 sessions were excluded. Participants were instructed to maintain a sleep diary upon returning home.

The inclusion criteria were as follows: (1) a diagnosis of chronic insomnia (for more than 3 months) according to the Diagnostic and Statistical Manual of Mental Disorders, fourth edition; (2) age ≥ 18 years and non-perimenopausal women; (3) PSQI > 6 points [27]; (4) patients currently taking insomnia medication (they did not need to discontinue their medication, but their dose remained stable for 1 month before the experiment); (5) a neurological examination revealing no positive findings; (6) accessible audio-visual equipment to complete the questionnaires and examinations required for the study; and (7) an informed consent form signed by the patient or their family member. Exclusion criteria were as follows: (1) patients with a history of other mental illnesses, alcohol or drug abuse or dependence, or low intelligence; (2) clinical evidence of neurological or other physical diseases, including respiratory, cardiac, renal, hepatic, and endocrine disorders; (3) women who were pregnant or breastfeeding; (4) patients undergoing psychotherapy or counseling concurrently; (5) insomnia caused by other organic diseases; and (6) medical conditions that, in the investigator’s opinion, precluded participation in the study.

### 2.2. Data Collection

Sociodemographic and clinical data were collected from patients, including age, sex, education level (coded as 1, illiterate; 2, primary school; 3, junior high school; 4, senior high school; 5, junior college; 6, bachelor’s degree; and 7, master’s degree), insomnia medication use and duration, and disease duration (number of years since diagnosis). We excluded illiterate patients to improve cooperation and included only those with primary or higher education. Moreover, the participants completed the study records and provided informed consent.

### 2.3. BFB Treatment

We conducted the BFB treatment at Xuanwu Hospital using BioNeuro Infiniti Bio 3000C V6.0.3 (T.T. Thought Technology Co., Montreal, QC, Canada). The eight-channel ProComp Infiniti encoder was used to collect data. The EEG signal was sampled at 256 Hz, with a bandpass of 0.5–70 Hz and a time constant of 0.3. The reference and ground electrodes were placed in the binaural mastoid with a pair of ear clips, and the impedance values were adjusted to below 5 kΩ. The signal electrodes of the EEG sensor were placed at the Cz point according to the International 10/20 System [28]. The EMG sensor was attached to the forehead with a headband.

**Figure 2:**
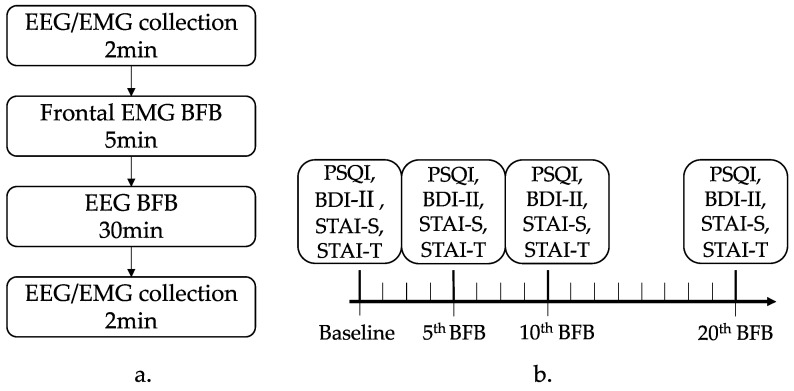
Experimental program schematic. (**a**) Biofeedback treatment flow chart for one session. (**b**) All process sketches including biofeedback treatment sessions and scale assessments. BDI-II, Beck Depression Inventory; BFB, biofeedback; EEG, electroencephalography; EMG, electromyography; PSQI, Pittsburgh Sleep Quality Index; STAI-S/T, State-Trait Anxiety Inventory.

Before and after the BFB treatment, EEG/EMG power was measured for 2 min while the participants were at rest. A 5 min period of decreasing frontal EMG BFB was followed by a 30 min period of increasing alpha power (8–12 Hz) neurofeedback training. Ten BFB sessions lasted 1 month and were conducted on Mondays, Wednesdays, and Fridays during the first and third weeks, and on Tuesdays and Thursdays during the second and fourth weeks. To avoid movement artifacts, participants were instructed to remain as still as possible during the experiment. All participants received the same treatment (Figure 2).

We adjusted the feedback thresholds based on the baseline measures at each session using the following formulae [26]: reinforcer: alpha mean amplitude (standard deviation/4); and inhibitor: EMG mean amplitude (standard deviation/2). All treatment sessions were conducted by the same experienced supervisor. During treatment, the therapist could adjust the threshold artificially and ensure that rewards were administered at 50–80% of the baseline [29]. A reward percentage was determined by the therapist and adjusted according to the participant’s motivation. To ensure participants were retained in the study, more feedback was provided if they were not motivated to complete all the neurofeedback sessions. For example, when training to increase alpha, if their alpha power was above the threshold, i.e., when the patient was relaxed, they would continuously hear beautiful music and watch beautiful scenery videos. The music and videos would stop if the alpha power fell below the threshold. At that point, participants needed only to adjust their own state of being and wait until the body and brain were completely relaxed. As the alpha power increased, the participant would resume hearing the music and viewing the video. If the therapist set the threshold too high and the patient struggled to reach it, their motivation was greatly reduced, especially if the patient was very anxious and could not concentrate long enough to complete the therapy. If the threshold was set too low, the patient could easily maintain motivation and the point of the therapy was negated. Video and audio playback were also important; a noisy video was not conducive to maintaining a relaxed and calm mood, and a lagging video led to patient irritation.

### 2.4. Evaluation

Primary outcome: EEG alpha power, beta power, theta power, and prefrontal EMG power were recorded before and after each BFB treatment session. Secondary outcome: sleep conditions (PSQI), depression severity (BDI-II), and anxiety severity (STAI) were assessed at baseline and after the fifth, tenth, and twentieth treatments [22]. In addition, the Treatment Emergent Symptom Scale and the Adverse Event Scale were completed.

PSQI is a self-rated questionnaire that assesses sleep quality and disturbances over a l-month time interval. Nineteen individual items generate seven “component” scores: subjective sleep quality, sleep latency, sleep duration, habitual sleep efficiency, sleep disturbances, use of sleeping medication, and daytime dysfunction. Each item is scored on a scale from 0 to 3 points, and the total score ranges from 0 to 21 points. PSQI > 6 points reflects poor sleep quality [27]. The Cronbach’s α coefficient for internal consistency of the total PSQI score was 0.84, which showed high reliability [30].

The revised BDI-II [31] is a widely used measure for assessing the severity of depression in psychiatric patients and for screening for possible depression in normal populations, according to the DSM-IV criteria for the diagnosis of depressive disorders. The BDI-II is scored by summing the highest ratings for each of the 21 items. Each item is rated on a 4-point scale ranging from 0 to 3, and the total scores can range from 0 to 63. BDI-II total scores of 0 to 13 indicate “minimal” depression; totals of 14 to 19 indicate “mild” depression; totals of 20 to 28 indicate “moderate” depression; and totals of 29 to 63 indicate “severe” depression. The BDI-II proved to be internally consistent (Cronbach’s α = 0.840) [32].

The STAI has 40 items, with 20 items each for the State-Trait Anxiety Inventory–State (STAI-S) and State-Trait Anxiety Inventory–Trait (STAI-T). The STAI-S assesses the current state of anxiety by asking respondents how they feel “right now” using items that measure subjective feelings of apprehension, tension, nervousness, worry, and autonomic nervous system activation/arousal. The STAI-T assesses relatively stable aspects of “anxiety proneness,” including general states of calmness, confidence, and security. Internal consistency alpha coefficients are quite high, ranging from 0.86 for high school students to 0.95 for military personnel [33]. The Cronbach’s α for the STAI-S was 0.950 and for the STAI-T was 0.926 in previous studies [34].

### 2.5. Data Preprocessing

Real-time online processing and display of results for the EEG and EMG signals were acquired using the amplifier. Unit time segmentation of the acquired signals was performed. Signal processing and analysis were performed in each segment. First, a 50 Hz trap was applied to the signal to filter out the industrial frequency noise, and then high- and low-pass filtering (0.5–45 Hz) was applied to remove unwanted signals using an infinite impulse response filter. Wavelet transform was performed on the pre-processed signal to decompose the alpha signal from 8 to 13 Hz and calculate the mean value. EMG signals were similarly captured, with high- and low-pass filtering per unit of time, and then averaged to calculate the display of real-time results [35,36,37].

### 2.6. Analysis

Data regarding age, disease course, educational background, and baseline scores (PSQI, BDI-II, and STAI-S/T) were analyzed using Wilcoxon rank-sum tests. Sex and insomnia medication use was analyzed using chi-square analysis. Linear mixed-effects models (LMMs) were used to analyze the PSQI, BDI-II, and STAI-S/T scores at baseline and after the fifth, tenth, and twentieth sessions. To assess the effect of the intervention over time, LMMs were used for repeated measures data. When repeated measures data were missing at random, LMMs provided accurate parameter estimates. Four time points were considered in the fitting of the models (baseline and after the fifth, tenth, and twentieth sessions).

As the primary outcome of our analysis of EEG alpha, beta, theta, and frontal EMG power, we defined the time coefficient as a measure of the effect of BFB over time and analyzed the results using analysis of repeated variance measures. The “post- vs. pre-treatment” coefficient represents the difference between before and after treatment, or the immediate effect, and the “time×treatment” coefficient describes the long-term trend. The change in EMGs was defined as the difference between the first EMG and the last EMG after treatment. The changes in scale (PSQI/BDI-II/ STAI-S/T) scores were defined as the first measured scale score minus the last measured scale score. Pearson correlation analyses were conducted for these two variables. Statistical analysis was conducted with SPSS Statistics for Windows, version 26.0. Differences were regarded as statistically significant at *p* ≤ 0.05.

## 3. Results

### 3.1. Clinical Characteristics

In total, 82 individuals completed more than 10 BFB sessions, including 14 individuals who completed more than 20 sessions. The average age of the participants was 49.38 ± 12.78 years, with 26 men and 56 women. There was no statistically significant difference between the 14 individuals who completed 20 sessions and the remaining 68 individuals in terms of demographic characteristics such as sex, age, education, and pre-treatment baseline scale scores (Table 1).

### 3.2. Sleep Scale (PSQI)

**Figure 3:**
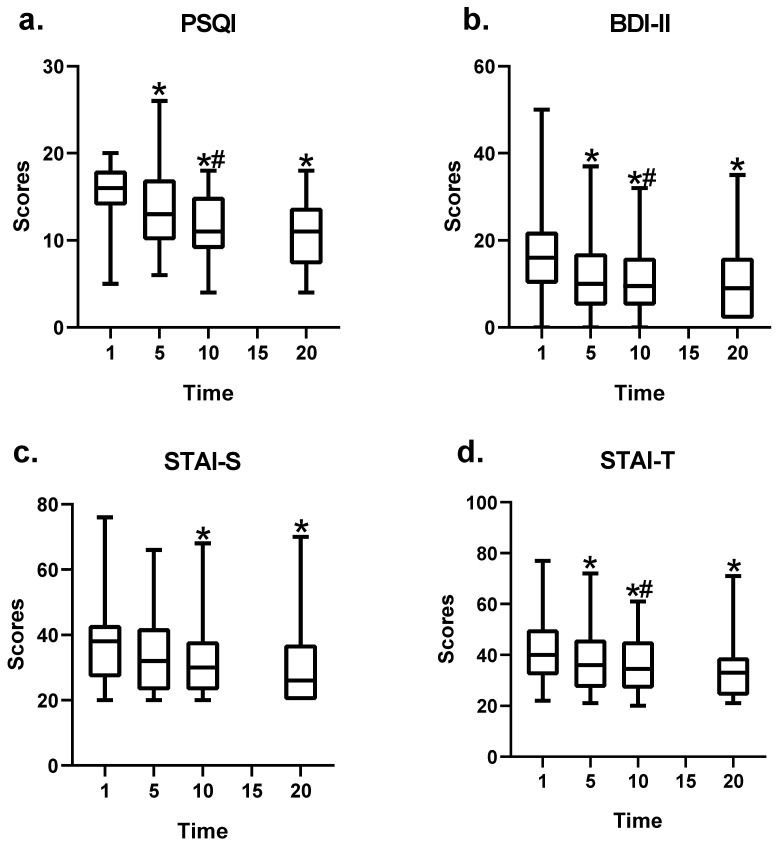
Changes in clinical scale scores before and after biofeedback sessions. (**a**) The PSQI scores before treatment and after the 5th, 10th, and 20th biofeedback sessions. (**b**) The BDI-II scores before treatment and after the 5th, 10th, and 20th biofeedback sessions. (**c**) The STAI-S scores before treatment and after the 5th, 10th, and 20th biofeedback sessions. (**d**) The STAI-T scores before treatment and after the 5th, 10th, and 20th biofeedback sessions. * Denotes a statistically significant difference from baseline (*p* < 0.05). # Denotes that the difference was statistically significant with comparison to the 5th BFB treatment sessions (median and Q1/Q3, *p* < 0.05). BDI-II, Beck Depression Inventory; PSQI, Pittsburgh Sleep Quality Index; STAI-S/T, State-Trait Anxiety Inventory.

PSQI scores after the 20th, 10th, and 5th BFB sessions were significantly lower than those before treatment (−5.50 ± 1.43, t = −3.85, *p* = 0.006; −3.64 ± 0.57, t = −6.4, *p* < 0.001; and −2.00 ± 0.51, t = −3.99, *p* < 0.001, respectively). The PSQI score was significantly lower after the 10th session than after the 5th session (−2.42 ± 0.60, t = −4.03, *p* < 0.001). There was no statistical difference in the PSQI scores between the 20th and 5th sessions (*p* =0.107) and between the 20th and 10th sessions (*p* = 0.479) (Figure 3a).

### 3.3. Emotional Scales

#### 3.3.1. BDI-II

BDI-II scores were significantly lower after the 20th, 10th, and 5th sessions than before treatment (−7.15 ± 2.43, t = −2.94, *p* = 0.012; −6.1 ± 0.71, t = −8.55, *p* < 0.001; and −4.96 ± 0.64, t = −7.71, *p* < 0.00, respectively). BDI-II scores were significantly lower after the 10th session than after the 5th session (−1.79 ± 0.53, t = −3.35, *p* = 0.001). Other comparisons were not statistically significant (Figure 3b).

#### 3.3.2. STAI (STAI-S and STAI-T)

STAI-S scores were significantly lower after the 20th and 10th sessions than before treatment (−12.36 ± 3.40, t = −3.63, *p* = 0.003; and −10.47 ± 4.78, t = −2.19, *p* = 0.031, respectively). Other comparisons did not show any significant differences (Figure 3c).

STAI-T scores were significantly lower after the 20th, 10th, and 5th sessions than before treatment (−9.86 ± 2.38, t = −4.41, *p* = 0.001; −5.57 ± 1.01, t = −5.53, *p* < 0.001; and −4.88 ± 0.93, t = −5.27, *p* < 0.001, respectively). STAI-T scores were significantly lower after the 10th session than after the 5th session (−2.09 ± 0.68, t = −3.07, *p* = 0.003). Compared with the 5th session, there was no significant difference in STAI-T scores between the 20th and 10th sessions (Figure 3d).

### 3.4. EEG

In the analysis of the two-factor repeated variance measures, there were two variables: the number of BFB treatments, with 20 levels (20 BFB sessions), and the treatment effect, with 2 levels (before and after treatment). According to the immediate post-treatment effect, post-treatment beta power significantly decreased compared with pre-treatment (F = 6.25, *p* = 0.014), and post-treatment theta power statistically decreased compared with pre-treatment (F = 11.91, *p* = 0.001). Over time after treatment, beta power decreased significantly (F = 2.02, *p* = 0.035), and theta power decreased significantly (F = 2.15, *p* = 0.024). There was no significant interaction between the two variables. Alpha power increased more after treatment than before treatment but the difference was not prominently significant (*p* > 0.05) (Table 2).

**Figure 4:**
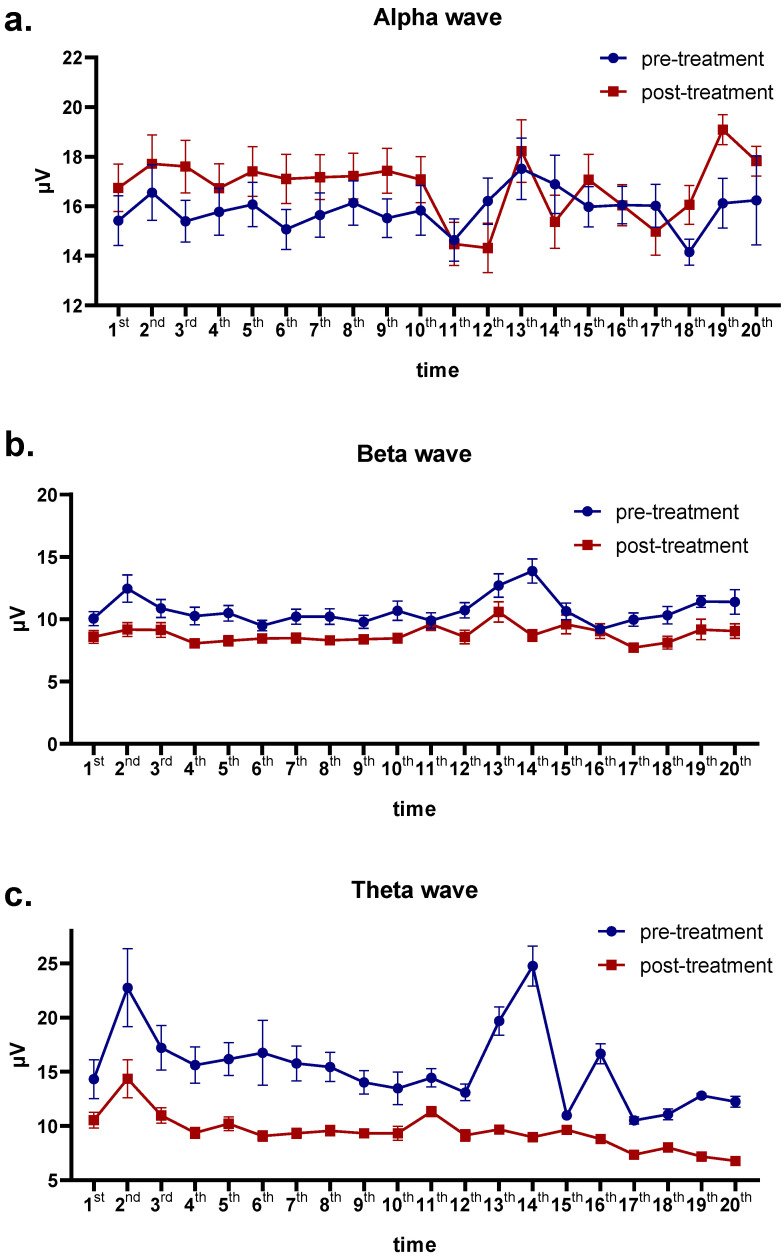
Changes in EEG waves pre- and post-treatment. (**a**) Alpha power before and after each of the 20 biofeedback sessions. (**b**) Beta power before and after each of the 20 biofeedback sessions. (**c**) Theta power before and after each of the 20 biofeedback sessions. Data presented as mean ± standard error. EEG, electroencephalography.

Patients with insomnia who received BFB treatment experienced increased activation intensity of the alpha frequency band and decreased activation intensity of the beta and theta frequency bands. As treatment time increased, this effect did not decrease or increase (Figure 4a–c). Alpha power increased significantly after each of the first 10 biofeedback sessions, with a trough after the 11th and 12th BFB sessions and a peak after the 19th and 20th sessions (that is, the maximum value of alpha over the whole course). Beta power decreased steadily with only slight fluctuations after the treatments, with a peak after the 13th BFB treatment session and a trough after the 17th and 18th BFB treatment sessions. Theta, on the other hand, decreased significantly after each BFB treatment session, with a peak after the 2nd treatment and a trough after the 20th treatment (that is, the minimum value of theta over the whole course).

### 3.5. EMG

We used a repeated variance measures linear model with two within-test variables: the number of treatments with 20 levels and the treatment effect with 2 levels (before and after treatment). The main effect of treatment was prominent (F = 2.11, *p* = 0.015). EMG tension level was significantly lower after treatment than before treatment. The duration of treatment had a significant effect (F = 2.91, *p* = 0.002). There was no significant interaction between the two variables (*p* > 0.05) (Table 2).

**Figure 5:**
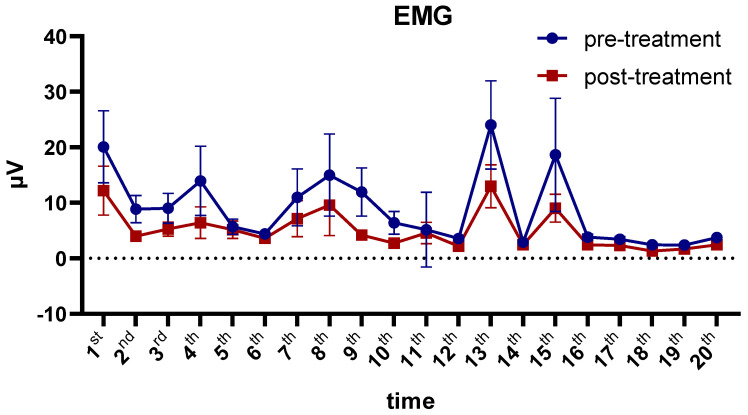
EMG power before and after each biofeedback session. EMG, electromyography. Data presented as mean ± standard error.

According to the study results, BFB treatment effectively decreased EMG activation in patients with sleep disorders. A longer treatment duration of more than 20 sessions may enhance this benefit (Figure 5).

### 3.6. Regression Analysis

#### 3.6.1. Reduced EMG Predicted Relief from Depression

**Figure 6:**
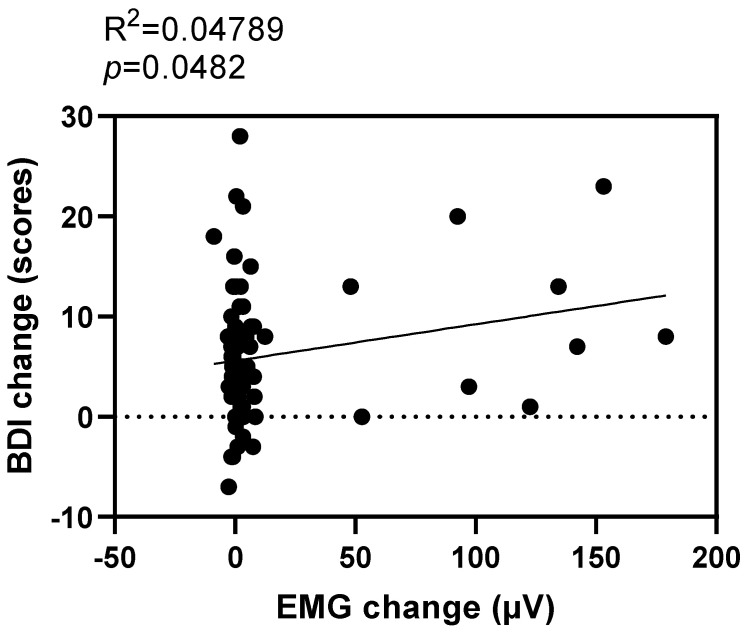
Regression results for EMG and BDI-II. The slash represents the slope. BDI-II, Beck Depression Inventory; EMG, electromyography.

Using the change in EMG as an independent variable and the change in BDI-II as the dependent variable, the regression analysis indicated that a decrease in EMG power could significantly predict a reduction in the BDI-II scores as an indicator of depression (r^2^ = 0.0478, *p* = 0.0482) (Figure 6).

#### 3.6.2. Symptom Duration Was Negatively Correlated with Trait Anxiety Response

**Figure 7:**
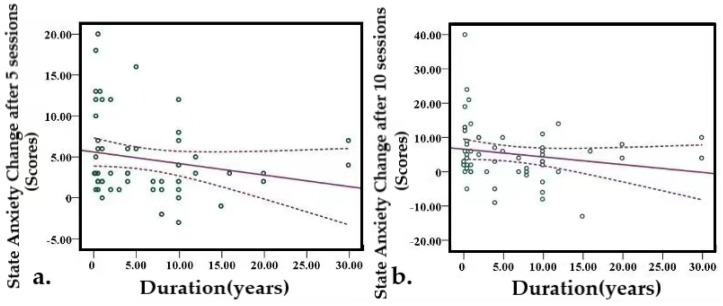
Correlation of insomnia duration and STAI. (**a**) State anxiety change after 5 sessions. (**b**) State anxiety change after 10 sessions. The dashed line represents the standard error and the slash represents the slope. STAI-S/T, State-Trait Anxiety Inventory.

Each patient’s illness lasted for a different length of time. The Pearson correlation analysis revealed that the longer the illness lasted, the smaller the decrease in state anxiety. A significant negative correlation was observed between symptom duration and state anxiety reduction after the 5th session (r = −0.325, *p* = 0.014) and after the 10th session (r = −0.395, *p* = 0.003) (Figure 7a,b).

## 4. Discussion

Our research demonstrated that by increasing alpha power and decreasing prefrontal EMG power, BFB treatment significantly reduced the symptoms of insomnia, anxiety, and depression in patients with insomnia, with noticeable improvements after 5 sessions of BFB treatment and further improvements after 10 or more sessions. To achieve a consistent treatment effect, a minimum of 10 sessions is required. A significant reduction in insomnia was observed, as was an easier transition to natural sleep, a shorter time to fall asleep, less waking while sleeping, and an improvement in the quality of sleep. After each BFB session, frontal EMG activation was almost always decreased. BFB sessions reduced EMG activity without it being affected by the number of treatments, suggesting that one session could reduce EMG activity and relieve muscle tension, regardless of whether the forehead muscles were tense before training. A decrease in frontal EMG predicted depression remission, and the longer the duration of insomnia, the fewer the anxiety symptoms alleviated by BFB treatment. According to previous research, progressive relaxation and EMG BFB significantly reduced sleep onset latency and depressive symptomatology, thus altering participants’ attentional processes and thereby reducing cognitive arousal [38]. The standard theory states that muscle relaxation reduces anxiety by creating a physical state that counteracts the fight-or-flight response. Muscle relaxation lowers and alters HR, blood pressure, and stress hormone levels [39]. We assume that the high pre-treatment frontal EMG activity decreases after BFB treatment, which is associated with relief from insomnia and anxiety–depression.

In addition, we found that the pre-treatment brain power frequencies band tended to be lower during alpha power and higher during beta and theta power and that increasing the alpha power with BFB treatment could reduce beta and theta power. During thought, concentration, attention, nervousness, alertness, and excitement, beta power (20–30 Hz) is generated [40]. A previous meta-analysis of EEG power during periods of wakefulness demonstrated that absolute beta power increases significantly and powerfully, and absolute theta power significantly increases [21]. Cortical hyperexcitability is observed as an increased high-frequency EEG amplitude in patients with insomnia [41]. Neurofeedback may alleviate insomnia symptoms by reducing cortical hyperarousal in patients [11]. Theta power (4–8 Hz) is generally observed during wakefulness or the first stage of sleep [25], and its enhancement is associated with sleepiness [42]. It has also been observed during depression, anxiety, and distraction [22,43]. Patients with insomnia experience elevated waking theta power, sleepiness, and impaired cognitive performance, as well as drowsiness and fatigue related to overnight sleep disruptions or hypnotics [44,45]. Alpha and theta amplitudes increase, while beta amplitude decreases in insomniac patients with depression [46]. For depression, addiction, and anxiety, alpha-increasing and theta-decreasing neurofeedback treatments are the most popular stress reduction strategies, increasing creativity and relaxation, improving musical performance, and healing trauma responses [47]. Researchers have documented that increased sensorimotor rhythm feedback (12–15 Hz), suppressed theta power, and increased beta power under Cz may alleviate insomnia [48]. Therefore, a combination of increased alpha and decreased beta and theta power may alleviate insomnia and anxiety/depression symptoms.

Moreover, our study demonstrated that the therapist played an essential role in BFB treatment. Specifically, frontal EMG activity, which is high in individuals with insomnia before treatment, is difficult to change through self-regulation. Under the guidance of a therapist, these patients were able to relax and maintain their relaxation within one or two sessions after receiving EMG feedback training. Cortoos’ remote BFB treatment of patients with insomnia without the presence of a trainer revealed little difference in frontal EMG power before and after treatment [26]. EEG feedback, in which therapists continuously adjust thresholds in real time according to a patient’s condition, is also an important factor in treatment effectiveness and patient cooperation.

In total, 121 patients with insomnia were treated with BFB, and 82 of them attended more than 10 sessions without experiencing any adverse effects. Most patients fell asleep during the BFB treatment and felt well afterward. Patients with insomnia also expressed a high level of acceptance of BFB treatment; once they have mastered the technique and succeeded in relaxing, they are willing to continue participating and developing positive sleep habits. In addition to building confidence in patients with insomnia who are reluctant to take prescribed medications, this study’s findings suggest that BFB may also improve sleep quality. The proportion of patients who completed all 20 sessions and had never taken insomnia medication was much higher than that of patients who completed only 10 sessions of BFB.

Our study has several limitations. First, patients were not followed, so it is unknown how long treatment efficacy can be maintained. Second, EEG BFB was restricted to a Cz single channel. Whole-brain EEG analysis, such as a topographic color map [11], could be used in the future to compare the changes in different brain power bands. Third, a sham BFB treatment as a control was not included in our study. Randomized controlled double-blind studies on BFB treatment are needed to define the effect of BFB treatment on insomnia. Lastly, the video graphics used in BFB treatment could be more varied and attractive to patients during follow-up sessions. Using brain–computer interface systems, adding new games [21], and using virtual reality devices may further reduce the attrition rate.

## 5. Conclusions

BFB treatment based on increasing alpha power and decreasing prefrontal EMG improves insomnia as well as anxiety and depression, with the symptom improvement becoming more pronounced and stable after more than 10 sessions. Biofeedback treatment that increases alpha power can effectively reduce beta and theta power, reducing alertness and promoting deep sleep. In addition, biofeedback treatment was effective in decreasing prefrontal EMG activation in patients with sleep disorders, and a reduction in prefrontal EMG can also predict an improved mood state. We will continue to control for other variables and conduct more extensive whole-brain EEG power analyses to explore the changes and effects produced by BFB treatment or combinations of EEG analyses at different locations and frequencies.

## Figures and Tables

**Table 1:** Demographic and clinical characteristics of the participants.

Variables ^a^	10–20 BFB Sessions (*n* = 68)	>20 BFB Sessions (*n* = 14)	*X* ^2^ */z*	*p*-Value ^b^
Sex (female/male)	46/22	10/4	0.094	0.158
Medication use (yes/no)	22/46	3/11	0.209	0.259
Age (years)	49.45 ± 13.24	48.95 ± 10.02	−0.813	0.416
Education (years)	13.63 ± 4.76	12.36 ± 6.08	−0.554	0.580
Duration (years)	5.29 ± 3.285	4.36 ± 1.99	−0.876	0.387
PSQI	14.77 ± 3.63	11.00 ± 4.41	−0.165	0.869
BDI-II	16.19 ± 8.12	10.85 ± 10.17	−0.751	0.453
STAI-S	42.22 ± 49.21	32.22 ± 14.88	−1.688	0.091
STAI-T	41.23 ± 11.21	35.86 ± 13.09	−0.341	0.733

^a^ Data are presented as mean ± standard deviation. ^b^ The *p*-value was obtained using a two-sample, two-tailed *t*-test. BDI-II, Beck Depression Inventory; BFB, biofeedback; PSQI, Pittsburgh Sleep Quality Index; STAI-S/T, State-Trait Anxiety Inventory.

**Table 2:** Improvement in the final EEG and EMG power compared with baseline after BFB treatment.

Item	Baseline ^a^ (Mean ± SD)	After 20 Sessions ^a^ (Mean ± SD)	Effect	df	F	*p*-Value ^b^
Alpha	15.42 ± 9.07	16.24 ± 7.09	Time	9	0.95	0.483
			post- vs. pre-treatment	1	0.11	0.736
			Time × treatment	9	0.99	0.450
Beta	10.06 ± 5.07	9.06 ± 5.27	Time	9	2.02	0.035
			post- vs. pre-treatment	1	6.25	0.014
			Time × treatment	9	0.64	0.764
Theta	14.31 ± 10.53	6.76 ± 1.07	Time	9	2.15	0.024
			post- vs. pre-treatment	1	11.91	0.001
			Time × treatment	9	0.27	0.981
EMG	20.08 ± 58.73	2.44 ± 1.15	Time	9	2.91	0.002
			post- vs. pre-treatment	1	2.11	0.015
			Time × treatment	9	0.36	0.952

^a^ The mean power of EEG and EMG are presented as mean ± standard deviation (SD) (µV). ^b^ The *p*-value was obtained by repeated measures analysis of variance. EEG, electroencephalography; EMG, electromyography.

## Data Availability

The data that support the findings of this study are available on request from the corresponding author. The data are not publicly available due to privacy or ethical restrictions.
